# Gene Expression Analysis of Four Radiation-resistant Bacteria

**DOI:** 10.4137/gei.s2380

**Published:** 2009-06-16

**Authors:** Na Gao, Bin-Guang Ma, Yu-Sheng Zhang, Qin Song, Ling-Ling Chen, Hong-Yu Zhang

**Affiliations:** 1 Shandong Provincial Research Center for Bioinformatic Engineering and Technique, Center for Advanced Study, School of Life Sciences, Shandong University of Technology, Zibo 255049, P.R. China.; 2 Computational Biology Unit, Bergen Center for Computational Science, University of Bergen, Bergen 5008, Norway.

**Keywords:** radiation-resistant bacteria, predicted highly expressed (PHX) genes, *E* value, recombinase A, antioxidant enzymes

## Abstract

To investigate the general radiation-resistant mechanisms of bacteria, bioinformatic method was employed to predict highly expressed genes for four radiation-resistant bacteria, i.e. *Deinococcus geothermalis* (*D. geo*), *Deinococcus radiodurans* (*D. rad*), *Kineococcus radiotolerans* (*K. rad*) and *Rubrobacter xylanophilus* (*R. xyl*). It is revealed that most of the three reference gene sets, i.e. ribosomal proteins, transcription factors and major chaperones, are generally highly expressed in the four bacteria. Recombinase A (*recA*), a key enzyme in recombinational repair, is predicted to be highly or marginally highly expressed in the four bacteria. However, most proteins associated with other repair systems show low expression levels. Some genes participating in ‘information storage and processing,’ ‘cellular processes and signaling’ and ‘metabolism’ are among the top twenty predicted highly expressed (PHX) genes in the four genomes. Many antioxidant enzymes and proteases are commonly highly expressed in the four bacteria, indicating that these enzymes play important roles in resisting irradiation. Finally, a number of ‘hypothetical genes’ are among the top twenty PHX genes in each genome, some of them might contribute vitally to resist irradiation. Some of the prediction results are supported by experimental evidence. All the above information not only helps to understand the radiation-resistant mechanisms but also provides clues for identifying new radiation-resistant genes from these bacteria.

## Introduction

Radiation-resistant bacteria can withstand desiccation and many kinds of radiations such as ultraviolet light (UV), Hg(II)-, U(VI)-, Cr(VI)-ionizing radiations (IR), Co-gamma and UV-gamma radiations.^1^,^2^,^3^,^4^,^5^ Unraveling the radiation-resistant mechanisms is an intriguing topic in current life sciences. Thanks to the rapid progress in genome sequencing projects, the complete genomes of four radiation-resistant bacteria, i.e. *Deinococcus geothermalis* DSM 11300 (*D. geo*),^4^ *Deinococcus radiodurans* R1 (*D. rad*),^1^ *Kineococcus radiotolerans* SRS30216 (*K. rad*)^3^,^5^ and *Rubrobacter xylanophilus* DSM 9941 (*R. xyl*),^2^ have been sequenced, which provide an unprecedented opportunity for exploring the radiation-resistant mechanisms of these bacteria.

The above four radiation-resistant aerobic bacteria survive in an intense dosage of radiation that is lethal to most organisms.^6^ The complete genome of *D. geo* is composed of a circular chromosome and two megaplasmids, which was originally isolated from a hot spring, and subsequently identified from other extreme environments.^7^,^4^ *D. rad* is the first deinobacteria discovered and isolated from canned meat that had spoiled following exposure to X rays, which contains two circular chromosomes and two plasmids.^1^ The above two bacteria belong to extremely radiation-resistant family *Deinococcus*, which can survive acute exposures to IR (10 kGy), UV (1 kJ/m^2^), and can grow under chronic IR (60 Gy/hour).^8^–^10^ *K. rad* contains a 4.76 Mb linear chromosome and two plasmids, which is isolated from a high-level radioactive environment.^3^,^5^ The thermophilic bacteria *R. xyl* contains a circular chromosome.^2^ Both *K. rad* and *R. xyl* belong to Actinobacteria phylum, which can withstand relatively high concentration of metals and alkali cations, as well as exposure to extreme doses of IR close to that of *D. rad*.^2^,^3^,^5^ The genome size, G+C content, optimal growth temperature (OGT) and other features of the four radiation-resistant bacteria are listed in Table 1.

**Figure 1:**
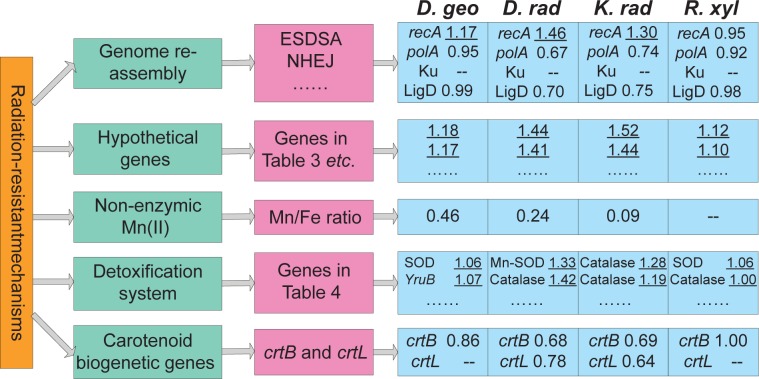
Illustration of the prevailing opinions of radiation-resistant mechanisms, some related genes and their predicted *E*(*g*) values in the four radiation-resistant bacteria. The Mn/Fe ratio is from Daly et al.^17^

Briefly, the prevailing opinions of radiation-resistant mechanisms are: (i) Genome reassembly and DNA repair are facilitated by numerous repeated sequences and morphology features, extended synthesis-dependent strand annealing (ESDSA) repair, nonhomologous end joining (NHEJ) repair and other DNA repair systems.^5^,^11^–^14^ (ii) A large number of ‘hypothetical genes’ might encode proteins related to unexplored radiation-resistant mechanisms.^15^ (iii) Accumulation of non-enzymic Mn(II) complexes help to protect proteins from oxidation during irradiation, and elemental ratios of Mn/Fe have been proposed to indicate a cell’s susceptibility to oxidative stress.^16^,^17^ (iv) Conventional enzymatic detoxifying systems might operate with extraordinary efficiency.^13^,^17^ (v) Genes involved in carotenoid biogenesis can confer a modest level of radiation resistance by scavenging reactive oxygen species (ROS).^18^,^19^ The mechanisms and some related genes are illustrated in Figure 1. However, no single opinion can explain the underlying genetic complexity of the extreme resistance phenotype.

To further understand the radiation-resistant mechanisms of these bacteria, a viable strategy is to identify the highly expressed genes. Thanks to the rapid progress in bioinformatics, several theoretical indices have been proposed to predict the expression levels of prokaryotic genes, which include codon adaptation index (CAI),^20^ effective number of codons,^21^ frequency of optimal codons,^22^ *E*(*g*) index^23^–^26^ *etc*. Based on *E*(*g*) index, Karlin and coworkers predicted the gene expression level of *D. rad*.^25^ It was found that a high number of chaperone/degradation, protease, detoxification genes, and several proteins of cell envelope surface structures are highly expressed. Many cell division proteins, ABC transporter genes and a high number of function-unknown genes are also predicted to be highly expressed.^25^ The abundance and versatility of the above predicted highly expressed (PHX) genes help to maintain the survival of *D. rad* when exposed to severe conditions of radiations. However, it remains unclear whether these mechanisms are also shared by other radiation-resistant bacteria, which stimulated our interest to do a comprehensive analysis. Considering the validity of *E*(*g*) based gene expression prediction methodology,^23^–^26^ it is employed in the present work to perform our analysis.

## Results and Discussion

### Statistics of PHX genes among the four radiation-resistant bacteria

The statistics of PHX genes and their *E*(*g*) values for the four radiation-resistant and one radiation-sensitive bacteria is listed in Table 2. As is shown, the percentage of PHX genes account for 9.6% to 14.1% of all the genes in the four radiation-resistant bacteria, and 19.1%∼31.2% of the PHX genes are function-unknown ‘hypothetical genes’. However, the number of annotated and PHX ‘hypothetical genes’ in radiation-sensitive *Escherichia coli* str. K12 (*E. coli*) is much smaller than the four radiation-resistant bacteria. The highest *E*(*g*) value ranges from 1.17 in *R. xyl* to 1.66 in *D. rad*. Table 3 displays the top twenty PHX genes in the four radiation-resistant and bacteria, and their corresponding *E*(*g*) values in *E. coli*. The following section will analyze these PHX genes with details.

## The Top Twenty PHX Genes in the Four Radiation-resistant Bacteria

### The top twenty PHX genes in reference gene sets RPs, TFs, and CHs

It is observed that genes in three reference groups (RPs, TFs, and CHs) are generally highly expressed (Table 3). Many 50 S (L2, L3, L15, L19, L22, L24) and 30 S ribosomal proteins (S1∼S4, S13) are among the top twenty PHX genes in one or more radiation-resistant bacteria. Especially, 30S ribosomal protein S1 is among the top twenty PHX genes in the analyzed four genomes. In the TF group, the translation elongation factor G (*fusA*), elongation factor Tu, Ts, Crp/Fnr family transcriptional regulator, RNA polymerase beta and beta’ subunit are generally highly expressed in the four species, especially RNA polymerase beta’ subunit, which is among the top twenty PHX genes in three radiation-resistant bacteria. The principal transcription/translation factors ensure microbes to synthesize proteins rapidly and exactly to prevent radiation damage. Some major chaperone proteins, such as *DnaK* (HSP70), *DnaJ*, *GroEL* (HSP60) and co-chaperonin *GroES* are among the top twenty PHX genes in the four species. *DnaK* can cooperate with trigger factor in the *de novo* protein folding by transiently binding to nascent and newly synthesized polypeptides.^27^ *GroEL* and *GroES* chaperones consist of a nest with a lid, which provide secluded environments for folding molecules to protect them against intermolecular aggregation and facilitate the actual folding process.^28^ The high expression levels of *DnaK*, *GroEL* and *GroES* are helpful for preventing protein misfolding and increasing their thermal stability, which might be helpful for resisting irradiation.^29^,^30^

### The top twenty PHX genes in ‘information storage and processing’ and ‘cellular processes and signaling’

According to the clusters of orthologous groups of proteins (COGs) category,^31^ all the functional genes are classified into four functional groups, i.e. ‘information storage and processing’, ‘cellular processes and signaling’, ‘metabolism’ and ‘poorly character-ized’, respectively.^31^ In ‘information storage and processing’ functional category, translation-associated GTPase, recombinase A (*recA*), GCN5-related N-acetyltransferase and DEAD/DEAH box helicase domain-containing protein are top twenty PHX genes in one or more radiation-resistant bacteria. Among them, *recA* is a key enzyme in recombination and repair, which is highly expressed in three radiation-resistant bacteria and marginally highly expressed in *R. xylanophilus*. As a major defense against environmental damage to cells, DNA repair is present in all organisms. It is presumed that other proteins involved in DNA repair processes should also be highly expressed, whereas the prediction result is much different from our speculation. Both Karlin et al’s and our analyses showed that most genes involved in direct repair, base excision repair, apurinic/apyrimidinic (AP) endonuclease, mismatch excision repair, nucleotide excision repair, recombinational repair (except *recA*) and other repair proteins are not highly expressed (data not shown). Therefore, it is speculated that *recA* gene makes more contribution for resisting irradiations than other repair proteins. All basal DNA repair genes in radiation-resistant bacteria are subject to positive selection.^32^ It is reported that recombinational processes contribute vitally to reconstituting the cleaved DNA fragments.^25^ Furthermore, experimental evidence shows that *recA* in *D. rad* is substantially up-regulated at early phase of DNA damage,^33^ and mutations in *recA* render *D. rad* as sensitive to ionizing radiation as *E. coli*.^5^

Some genes in ‘cellular processes and signaling’ are among the top twenty PHX genes in the four genomes. However, most of them are not generally highly expressed (Table 3) except ATP-dependent protease and S-layer-like protein. ATP-dependent protease is described with details in the next section. Cell surface proteins can envelop the cell exterior, which provide protection against environmental desiccation, thermal effect and other hazards in the environment. S-layer protein (surface structure) is among the top twenty PHX genes in *D. geo* and *D. rad*, whereas the other two species have no similar genes.

### The top twenty PHX genes in ‘metabolism’ functional category

Many genes participating in ‘energy production and conversion’, ‘lipid metabolism’, ‘amino acid transport and metabolism’ and ‘nucleotide transport and metabolism’ are among the top twenty PHX genes and generally highly expressed in the four radiation-resistant bacteria. Aconitate hydratase (*acnA*) of tricarboxylic acid (TCA) cycle, antioxidant protein thioredoxin, F0F1 ATP synthase and oxidoreductase-like protein are usually highly expressed in the four radiation-resistant bacteria. Whereas, other genes, such as aldo/keto reductase, isocitrate lyase (*aceA*) in glyoxalate bypass and light-independent protochlorophyllide reductase subunit B, are PHX genes in one or two species. In ‘lipid metabolism’ category, only acyl-CoA dehydrogenase-like protein is highly expressed in three bacteria except *K. rad*. ABC transporter can transport a wide variety of substrates including sugars, amino acids, metal ions, peptides, proteins, and a large number of hydrophobic compounds and metabolites across extra- and intracellular membranes, which is essential for all living organisms.^34^ Some proteins in ‘amino acid transport and metabolism’ are commonly highly expressed in the four bacteria. However, a number of genes in ‘amino acid transport and metabolism’ category are highly expressed only in one or two genomes. For example, histidinol phosphate aminotransferase are the top twenty PHX genes in *R. xyl*, whereas it is not annotated in the other three bacteria. Some extracellular solute binding proteins are highly expressed in *D. geo* and *D. rad*. Furthermore, glycine hydroxymethyltransferase is only highly expressed in *K. rad*. Nucleoside-diphosphate kinase in ‘nucleotide transport and metabolism’ is highly expressed in three bacteria except *R. xyl*, and bifunctional 2′, 3′-cyclic nucleotide 2′-phosphodiesterase is highly expressed in *D. geo* and *K. rad*.

### The top twenty PHX genes with special and hypothetical function

A membrane lipoprotein is one of the top twenty PHX genes in *D. geo* and highly expressed in *D. rad* and *K. rad*. Furthermore, a large number of ‘hypothetical genes’ (ranging from 46 in *D. geo* to 145 in *K. rad*) are highly expressed in the four radiation-resistant species, and some of them are among the top twenty PHX genes (Table 3). Since ‘hypothetical genes’ account for 22.6%∼47.8% of the four radiation-resistant bacteria, some of them might be unexplored radiation-resistance proteins which are more effective than the known counterparts. Functions of these ‘hypothetical genes’ are of special interest for future studies.

### Comparing the top twenty PHX genes with radiation-sensitive bacterium *E. coli*

Comparing the top 20 PHX genes with well-studied radiation-sensitive bacterium *E. coli*, it could be observed that their expressions are much different (Table 3). Although the reference gene sets RPs, TFs and chaperones are highly expressed in *E. coli*, none of them are among the top twenty PHX genes. However, many of these reference genes belong to the top 20 highly expressed in the four radiation-resistant bacteria. The relative abundance of RPs, TFs and chaperones might help protein synthesis and folding more efficiently in radiation-resistant bacteria. Some tRNA synthetases are among the top 20 PHX genes, whereas none of them are among the top highly expressed in the four radiation-resistant bacteria. Furthermore, most top PHX genes in radiation-resistant bacteria listed in Table 3 are not highly expressed in *E. coli*, indicating that gene expression patterns are much different in radiation-sensitive and radiation-resistant bacteria.

## PHX Genes in Antioxidant System and Proteolysis

Because the formation of ROS during irradiation is extremely rapid, the antioxidant enzymes in radiation-resistant bacteria must be highly efficient in order to neutralize and remove free radicals and other toxic substances. Many antioxidant enzymes are highly expressed in the four radiation-resistant bacteria (Table 4). For example, Cu-Zn superoxide dismutase (*sodC*) and Mn superoxide dismutase (*sodA*) are highly expressed in three radiation-resistant species except *K. rad*. Many catalase and peroxidase participating in detoxification are generally highly expressed in the four species. Thioredoxin reductase (*TrxR*) in conjunction with thioredoxin (*Trx*) is a ubiquitous oxidoreductase system with antioxidant and redox regulatory roles.^35^ Glutaredoxin is another important thiol-based antioxidant with function overlapping that of thioredoxin.^35^ Some thioredoxin and glutaredoxin related genes are commonly highly expressed in the four radiation-resistant bacteria. The multiple PHX detoxification genes help remove free radicals generated by irradiation. In addition, a vanadium-dependent haloperoxidase is highly expressed in *D. geo*, whereas the other three bacteria do not have this enzyme.

Some proteases participating in protein degradation are highly expressed in three radiation-resistant bacteria except *R. xyl*. Most highly expressed pro-teases are ATP-dependent proteases. ATP-dependent proteases control diverse cellular processes by degrading specific regulatory proteins. Especially, ATP-dependent protease La is essential for cellular homeostasis by mediating the degradation of abnormal and damaged polypeptides, as well as short-lived regulatory proteins.^36^ Furthermore, two serine proteases are also highly expressed in *D. rad*. High expression levels of these proteases make sure that the irradiation injury proteins could be degraded efficiently.

## Comparing the *in silico* Prediction Result with Experimental Evidence

**Figure 2:**
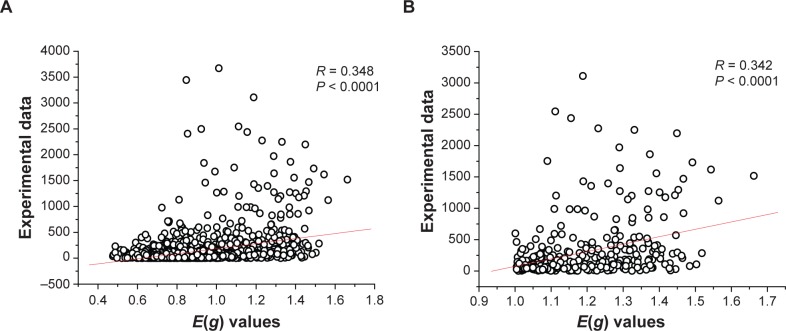
The correlation between predicted *E*(*g*) values and experimentally determined transcriptional profiling in wild-type *D. rad*. (**A**) Correlation of all the annotated genes. (**B**) Correlation of the PHX genes.

To evaluate the prediction reliability, *E*(*g*) values should be compared with experimental evidence. Although the four radiation-resistant bacteria have been sequenced for several years, only *D. rad* transcriptional profiling can be obtained in gene expression databases up to now. The experimental gene expression data of *D. rad* were downloaded from NCBI GEO database (http://www.ncbi.nlm.nih.gov/geo/query/acc.cgi?acc=GSE9636). Then the correlation between experimental and prediction result is analyzed. Figure 2 (a–b) show the correlation between *E*(*g*) values and experimental transcriptional profiling in wild-type *D. rad*. Figure 2 (a) is the correlation of all the annotated genes (correlation coefficient *R* = 0.348, *P* < 0.0001), and (b) shows the correlation of PHX genes and their experimental expression levels (*R* = 0.342, *P* < 0.0001). Although the correlation is weak, it is statistically significant. Further, Joshi et al reported the kinetics of proteomic changes accompanying post-irradiation recovery (PIR) in *D. rad* following exposure to 6 kGy γ-irradiation.^37^ Many PHX genes, such as a serine protease and some ‘hypothetical genes’ were found to be expressed at high levels and not degraded during long time of irradiation. Some PHX gene, such as aconitate hydratase (*acn*), *groEL*, *dnaK* and some ‘hypothetical genes’ were degraded during 4 h lag period of PIR and re-synthesized to normal levels during the late phase of PIR.^37^ Like DNA degradation, export and repair, degradation and resynthesis of damaged proteins can ensure rapid and smooth post-irradiation recovery of *D. rad*.^37^ The above examples show that the prediction results are in accordance with experimental evidence. In the absence of experimental data, the *in silico* prediction can provide some valuable information.

## Conclusion

Through analyzing the common PHX genes in four radiation-resistant bacteria, it can be inferred that some common radiation-resistant mechanisms are shared by the four species (Fig. 1). Our prediction and experimental evidence supports the opinion that *recA* is more important than other repair proteins in resisting irradiation. Furthermore, many antioxidant enzymes, e.g. superoxide dismutase, catalase, peroxidase and thiol-based thioredoxin and glutaredoxin, play important roles in scavenging free radicals caused by irradiation, which are commonly or uniquely PHX genes in the four radiation-resistant bacteria. Proteases are important for degrading abnormal and damaged polypeptides, some of them, especially ATP-dependent protease La, are PHX genes in most radiation-resistant bacteria. In addition to the common highly expressed genes, each species has its own unique PHX genes to resist irradiation. Another important issue is that a number of ‘hypothetical genes’ are PHX genes in the four radiation-resistant bacteria, and some are among the top twenty, which might be unexplored genes for resisting irradiation. All the above information is helpful for further understanding the radiation-resistant mechanisms and provides important clues to identifying new radiation-resistant genes.

## Materials and Methods

The complete sequences and corresponding annotation information of the four radiation-resistant bacteria *D. geo* (chromosome: NC_008025), *D. rad* (chromosome 1: NC_001263; chromosome 2: NC_001264), *K. rad* (NC_009664), *R. xyl* (NC_008148) and one radiation-sensitive model bacterium *E. coli* (NC_000913) were downloaded from NCBI RefSeq at Aug 6, 2008.

As a well-established formulism, Karlin’s methodology select three sets of genes, i.e. ribosomal proteins (RP), principal transcription/translation factors (TF), and the major chaperones/degradation (CH), as the reference of tacit highly expressed genes. Qualitatively, if a gene’s codon usage is similar to that of the three groups of genes but deviates strongly from the average gene of the genome, it is predicted to be a PHX gene.^23^–^26^ The *E* values can reflect the levels of common genes’ similarity in codon frequencies to RP, TF, and CH groups and of their deviation from the whole genome.

Let *F* and *G* be two groups of genes, the codon usage difference of *F* relative to *G* is calculated by the formula

$$B(F|G)=\sum_{a}p_{a}(F)\left[\sum_{(x,y,z)=a}|f(x,y,z)-g(x,y,z)|\right],$$ (1)

where *p_a_* (*F*) are the average amino acid frequencies of the genes in *F*, and *f*(*x*, *y*, *z*) and *g*(*x*, *y*, *z*) are the average codon frequencies for the codon triplet (*x*, *y*, *z*) of each amino acid codon family for gene groups *F* and *G*, respectively. Let *B* (*g*|*G*) indicates the codon usage difference of the gene *g* relative to the gene group *G*, and *C* is the totality of all genes in the genome.^25^ Predicted expression levels with respect to individual standards are based on the following

$$\begin{array}{l} E_{\mathrm{RP}}(g)=\frac{B(g|C)}{B(g|\mathrm{RP})},\\ E_{\mathrm{CH}}(g)=\frac{B(g|C)}{B(g|\mathrm{CH})},\\ E_{\mathrm{TF}}(g)=\frac{B(g|C)}{B(g|\mathrm{TF})}. \end{array}$$ (2)

For the four bacterial genomes, the overall expression measure is

$$E=E(g)=\frac{B(g|C)}{\frac{1}{2}B(g|\mathrm{RP})+\frac{1}{4}B(g|\mathrm{CH})+\frac{1}{4}B(g|\mathrm{TF})}.$$ (3)

PHX genes must satisfy the following two criteria, the *E* value exceeds 1.00 and at least two of *E*_RP_(*g*), *E*_CH_(*g*), and *E*_TF_(*g*) are more than 1.05.

## Figures and Tables

**Table 1:** General features of the four radiation-resistant bacteria.^a^

	*D. geo*	*D. rad*	*K. rad*	*R. xyl*
Chromosome Ac. No.	NC_008025	NC_001264	NC_009664	NC_008148
Genome size (bp)	3041332	3284156	4761183	3225748
G+C (%)	66.6	67.0	74.2	70.5
No. of Chromosome + plasmid	1 + 2	2 + 2	1 + 2	1 + 0
OGT^b^ (°C)	50	30	30	60
Radiation dosage^c^	10 kGy	16 kGy	20 kGy	5.5 kGy
Radiation type	IR	IR	γ-radiation	IR

a Abbreviations in the five tables are as follows: D. geo, Deinococcus geothermalis DSM 11300; D. rad, Deinococcus radiodurans R_1_; K. rad, Kineococcus radiotolerans SRS30216 and R. xyl, Rubrobacter xylanophilus DSM 9941.

b O_G_T indicates optimal growth temperature.

c Radiation dosage is from Daly et al^17^; Bagwell et al^5^ and Sghaier et al.^32^

**Table 2:** Statistics of PHX genes for one radiation-sensitive and the four radiation-resistant bacteria.

	*D. geo*	*D. rad*	*K. rad*	*R. xyl*	*E. coli*
Number of annotated genes	2335	2629	4480	3140	4132
Number of PHX genes	241	362	632	301	444
Number of hypothetical PHX genes	46	113	145	62	29
Percentage of PHX genes	10.3%	13.8%	14.1%	9.6%	10.7%
Highest *E*(*g*) value	1.31	1.66	1.58	1.17	1.31

**Table 3:** The top twenty PHX genes in one radiation-sensitive and the four radiation-resistant species.^a^

Gene	*E*(*g*)				
	*D. geo*	*D. rad*	*K. rad*	*R. xyl*	*E. coli*
**RPs**					
L2 (*rplB*)	**1.20**	**1.37**	**1.27**	**1.08**	**1.17**
L3 (*rplC*)	**1.18**	**1.37**	**1.36**	**1.03**	**1.16**
L15(*rplO*)	(1.03)	**1.31**	**1.58**	(1.00)	**1.13**
L19 (*rplS*)	**1.18**	**1.40**	**1.32**	(1.01)	**1.12**
L22 (*rplV*)	**1.19**	**1.38**	(1.03)	**1.05**	(1.09)
L24 (*rplX*)	**1.16**	**1.28**	**1.56**	(1.00)	**1.13**
S1 (*rpsA*)	**1.31**	**1.45**	**1.54**	**1.11**	**1.13**
S2 (*rpsB*)	**1.11**	**1.47**	**1.38**	(1.00)	**1.15**
S3 (*rpsC*)	**1.20**	**1.37**	**1.36**	**1.04**	**1.15**
S4(*rpsD*)	**1.21**	**1.39**	**1.43**	**1.17**	**1.12**
S13 (*rpsM*)	**1.05**	**1.18**	**1.43**	**1.04**	**1.05**
**TFs**					
Translation elongation factor G (*fusA*)	**1.18**	**1.45**	**1.41**	(1.00)	**1.15**
Elongation factor Tu	**1.07**	**1.44**	**1.43**	(0.97)	**1.15**
	**1.08**	**1.44**	–	(0.97)	**1.14**
Elongation factor Ts	(1.02)	**1.52**	**1.22**	**1.03**	**1.15**
Transcriptional regulator, Crp/Fnr family	**1.18**	**1.11**	(0.96)	**1.03**	–
RNA polymerase beta subunit (*rpoB*)	**1.11**	**1.41**	**1.53**	(0.87)	**1.19**
DNA-directed RNA polymerase subunit beta’	**1.06**	**1.47**	**1.80**	**1.11**	–
**Chaperones**					
DnaK protein (*dnaK*)	**1.29**	**1.50**	**1.53**	(0.98)	**1.17**
Chaperone DnaJ (*dnaJ*)	**1.13** **1.04**	(0.93)	**1.28**	**1.09**	**1.13**
60 kDa chaperonin (*groEL*)	**1.30**	**1.66**	**1.54**	**1.05**	**1.15**
Co-chaperonin *GroES*	**1.06**	**1.15**	**1.29**	**1.04**	**1.08**
**Information storage and processing**					
Translation-associated GTPase	(0.91)	**1.30**	(0.84)	**1.10**	(0.96)
Recombinase A (*recA*)	**1.17**	**1.46**	**1.30**	(0.95)	**1.08**
GCN5-related N-acetyltransferase		(0.81)	**1.08**	**1.10**	–
DeaD/Deah box helicase domain-containing protein	**1.17**	–	**1.50**	(0.90)	–
**Cellular processes and signaling**					
Glycosyl transferase family protein	(0.86)	(0.76)	**1.25**	**1.10**	(0.88)
	(0.90)		**1.24**	**1.07**	(0.89)
			**1.24**	**1.05**	(0.81)
				**1.09**	(0.85)
					(0.80)
					(1.02)
					(1.02)
ATP-dependent protease	**1.21**	**1.31**	**1.13**	–	**1.06**
Peptidase s8 and s53, subtilisin, kexin, sedolisin	**1.26**	–	**1.05**	(0.92)	–
Flagellar motor switch protein FliG	–	–	**1.53**	–	(0.93)
S-layer-like protein region	**1.18**	**1.49** 1.45	–	–	–
Mandelate racemase/muconate lactonizing enzyme-like protein	(0.89)		**1.07**	**1.10**	–
UTP-glucose-1-phosphate uridylyltransferase	–	–	**1.43**	–	1.05
Histidine kinase	(0.93)	–	**1.43**	(0.91)	
**Metabolism**					
**Energy production and conversion**					
Aconitate hydratase (*acnA*)	(1.00)	**1.56**	**1.38**	**1.06**	(0.68)
Aldo/keto reductase	**1.18**	(0.98)	(0.67)	(1.00)	(0.82)
Thioredoxin	(1.01)	**1.11**	**1.10**	**1.09**	**1.09**
			**1.07**	**1.02**	(0.93)
Isocitrate lyase (*aceA*)	**1.09**	**1.55**	–	(0.98)	(0.98)
F0F1 ATP synthase subunit beta	**1.05**	**1.10**	**1.54**		**1.12**
F0F1 ATP synthase subunit alpha	–		**1.51**	**1.07**	**1.17**
FAD-dependent pyridine nucleotide-disulphide oxidoreductase	(1.03)	(0.80)	**1.54**	**1.00**	(0.73)
Light-independent protochlorophyllide reductase subunit B		–	–	**1.12**	
Oxidoreductase-like protein	**1.03**	**1.09**	**1.13**	**1.10**	
**Lipid metabolism**					
Acyl-CoA dehydrogenase-like protein	**1.16**	**1.54**	(0.82)	**1.06**	(0.87)
	**1.06**				
	**1.05**				
**Amino acid transport and metabolism**					
Extracellular solute binding protein, family 5	**1.16**	**1.47**	–	–	–
	**1.12**	**1.47**			
	**1.06**	**1.02**			
		**1.01**			
Histidinol phosphate aminotransferase	–	–	–	**1.11**	(0.92)
Glycine hydroxymethyltransferase	(1.00)	–	**1.42**	–	
Branched-chain amino acid ABC transporter, periplasmic amino acid-binding protein	–	**1.50** 1.46	–	**1.08**	–
ABC transporter related inner-membrane translocator	**1.13** **1.05**	**1.48**	**1.53** **1.47**	**1.05** **1.05**	–
	(0.87)	–	**1.43**	**1.01**	–
Binding-protein-dependent transport systems inner membrane component	**1.08**	–	**1.23**	**1.11**	–
	**1.02**		**1.08**	**1.10**	
**Nucleotide transport and metabolism**					
Nucleoside-diphosphate kinase (*ndk*)	**1.19**	**1.05**	**1.18**	(0.96)	**1.11**
Bifunctional 2′,3′-cyclic nucleotide 2′-phosphodiesterase/3′-nucleotidase periplasmic precursor protein	**1.18**	(0.85)	**1.22**	–	–
**Others**					
Membrane lipoprotein	**1.17**	**1.43**	**1.22**	(0.83)	**1.22**
Hypothetical protein	**1.18**	**1.44**	**1.52**	**1.12**	
	**1.17**		**1.44**	**1.10**	
				**1.10**	
				**1.10**	
				**1.09**	

a The numbers underlined indicate the top 20 PHX genes; numbers in parentheses indicate the corresponding genes are not PHX, ‘–’ denotes the gene is absent in the corresponding species.

**Table 4:** PHX antioxidant enzymes in the four radiation-resistant bacteria.

Species	Enzyme	PID	*E*(*g*)
*D. geo*	SOD	94984937	1.06
	Uncharacterised peroxidase-related	94985546	1.04
	Thioredoxin reductase	94985676	1.08
	Glutaredoxin-like protein, YruB	94985608	1.07
	Acid phosphatase/vanadium-dependent haloperoxidase related	94984807	1.14
*D. rad*	Mn-SOD	15806297	1.33
	Cu, Zn-SOD	15806556	1.20
	Cu, Zn-SOD (in chromosome 2)	15807868	1.09
	Catalase	15806994	1.42
	Catalase (in chromosome 2)	15807922	1.41
	Chloride peroxidase	15805817	1.09
	Organic hydroperoxide resistance protein	15806857	1.06
	Thioredoxin	15805968	1.11
	Glutaredoxin, putative	15807079	1.00
*K. rad*	Catalase	152964783	1.28
	Catalase	152964833	1.19
	Chloride peroxidase	152964099	1.19
	Glutathione peroxidase	152965215	1.06
	Thioredoxin	152964816	1.10
	Thioredoxin	152968443	1.07
	Thioredoxin reductase	152968442	1.07
	Glutaredoxin	152967298	1.09
	Glutaredoxin-like protein	152965146	1.08
	Glutaredoxin-like protein NrdH	152964771	1.08
*R. xyl*	SOD	108805705	1.06
	Catalase	108803526	1.00
	Thioredoxin	108804664	1.02
	Thioredoxin	108803844	1.09

**Table 5:** PHX proteases in the four radiation-resistant bacteria.^a^

Species	Enzyme	PID	*E*(*g*)
*D. geo*	ATP-dependent protease La	94984535	1.21
	Carboxyl-terminal protease	15806561	1.30
	ATP-dependent protease La	15805378	1.27
	Protease I	15806218	1.22
*D. rad*	ATP-dependent protease ATP-binding subunit	15806971	1.19
	Serine protease, subtilase family, C-terminal fragment	15807313	1.18
	serine protease, subtilase family	15806936	1.17
*K. rad*	ATP-dependent metalloprotease FtsH	152964507	1.32
	ATP-dependent protease ATP-binding subunit	152967458	1.13
	ATP-dependent Clp protease adaptor protein ClpS	152967709	1.10

a *R. xyl* has no PHX proteases.
